# Mechanical Properties of Recycled Concrete Incorporated with Super-Absorbent Polymer and Machine-Made Stone Powder under the Freeze-Thaw Cycle Environment

**DOI:** 10.3390/ma17205006

**Published:** 2024-10-13

**Authors:** Lingling Zhang, Ronggui Liu, Feifei Jiang

**Affiliations:** 1 School of Civil Engineering, Nantong Institute of Technology, Nantong 226000, China; zll18662854351@163.com; 2 Faculty of Civil Engineering and Mechanics, Jiangsu University, Zhenjiang 212013, China

**Keywords:** freeze–thaw cycle, combined incorporation of SAP and MSP, mechanical strength, linear regression

## Abstract

Recycled concrete incorporating additional super-absorbent polymer (SAP) and machine-made stone powder (MSP) was prepared using a two-factor, four-level orthogonal test. To enhance the frost resistance of recycled concrete and improve its mechanical properties, such as compressive and flexural strength, the prepared concrete underwent 200 freeze–thaw cycles. Before freeze–thaw cycles, the amount of SAP has a predominant influence on the mechanical properties of recycled concrete in comparison with MSP. After 200 cycles of freeze–thaw, the influence of MSP became more significant than that of SAP. Typically, the compressive strength and flexural strength exhibited a trend of initially increasing and then decreasing as the contents of SAP and MSP increased. The optimized recycled concrete was identified as S16M6, containing 0.16% SAP and 6% MSP, as demonstrated by the minimal strength loss after freeze–thaw cycles. This study also proposed a linear regression model for predicting the mechanical properties which offered valuable guidance for the engineering application of recycled concrete mixed with SAP under the freeze–thaw cycle environment.

## 1. Introduction

Concrete is extensively utilized in construction, including bridges, tunnels, buildings, embankments, and harbors, due to its cost-effectiveness and versatility [1,2,3]. Its indispensable role in urban construction has led to a high demand for concrete production. However, this production has significantly impacted resources, energy, and the environment, including issues related to the supply of raw materials and the recycling of waste concrete [4,5,6].

Among the components that constitute concrete, aggregate has a significant impact on the quality of concrete due to its high proportion (ca. 60–70%) [7,8,9]. Currently, there is a shortage in the supply of sandstone. Although these materials are not rare, over-exploitation has led to shortage of sandstone [10,11]. In order to cope with the problem of resource shortage, recycled concrete produced by recycling sandstone from construction waste has been proposed [12,13,14,15,16]. This approach not only addresses raw material shortages but also effectively manages waste concrete. Recycled concrete predominantly uses machine-made sand and recycled aggregate to replace natural sand and aggregate in common concrete [17,18]. Machine-made sand, with particles smaller than 4.75 mm, is produced from various rocks and construction wastes through mechanical processing [19,20]. The strength grade of common concrete is generally determined by the dust content in natural sandstone. The strength of recycled concrete is determined by the content of crusher dust in the machine-made sand. The crusher dust of machine-made sand basic ingredients is machine-made stone powder (MSP), with particles smaller than 0.075 mm [21,22,23,24].

The production of recycled concrete from tunnel slag, particularly tunnel slag generated from tunnel excavation, has been recognized as a sustainable approach for slag treatment and slag recycling [25,26,27,28]. Slag aggregate-based concrete (SABC), made from tunnel slag, has been successfully used in tunneling projects [25,29,30] and has improved seismic performance and reduced collapse [31,32]. However, to enhance the application of such concrete in cold regions, further study on the effects of MSP content on mechanical properties and frost resistance is necessary [33,34]. Under the coupling effect of external loads and freeze–thaw environment, concrete structures suffer from cracking, resulting in reduced stability of the structure. Therefore, several studies have focused on the effect of MSP on the strength of recycled concrete. However, the effect of MSP on strength and frost resistance of recycled concrete is not uniform [35,36,37,38]. Generally, the strength of recycled concrete first decreases and then increases as the MSP content increases, until a good strength is obtained at 10% [39]. However, some studies indicate that MSP contents of 5% and 8% are beneficial to the frost resistance of concrete [29]. Tan et al. [40] believed that mixing an appropriate amount of stone powder could improve the frost resistance of concrete. Most researchers have concluded that incorporating an appropriate amount of MSP can improve the mechanical properties and frost resistance of recycled concrete [25,41,42]. However, the optimal dosage has not been specifically provided. Although this represents a breakthrough in the research on recycled concrete, the solidity and fluidity of machine-made sand are more defective than that of natural sand. Furthermore, excessive incorporation of MSP leads to shrinkage cracking, strength reduction, and impaired frost resistance in the recycled concrete structure [35,43,44]. To overcome these shortcomings, superabsorbent polymer (SAP) is added to ordinary concrete to deal with issues such as low water-cement ratios and significant shrinkage during drying, enhancing concrete strength, shrinkage cracking, structural density, and frost resistance [45,46,47]. Therefore, this study innovatively proposes to add admixtures to recycled concrete, such as adding SAP to address issues like a low water-cement ratio, poor workability, and high drying shrinkage. However, the incorporation of SAP reduces mechanical properties and frost resistance of the structure through leaving internal cavities [48,49,50,51]. When the SAP proportion was 0.2%, the compressive strength of the structure was reduced to 10–12% [52]. In addition, the strength of the structure decreased rather than increased when SAP was incorporated at proportions of 0.1%, 0.2%, 0.3%, 0.4%, and 0.5% [53,54]. Some researchers have suggested that the SAP release of water allows the structure to be porous, leading to a reduction in structural strength [48,55,56]. Therefore, in order to ensure the better mechanical properties and frost resistance of recycled concrete, the content of MSP and SAP added in recycled concrete are worthy of further study.

This study aims to balance the strength and frost resistance of recycled concrete by adjusting MSP (0%, 3%, 6%, 9%) and SAP (0%, 0.08%, 0.16%, 0.24%) dosages. Using the orthogonal test method, the effects on compressive strength, flexural strength, and frost resistance were analyzed to determine the optimal combination of MSP and SAP. The findings offer valuable insights into enhancing the mechanical properties and frost resistance of recycled concrete.

## 2. Orthogonal Experiments

### 2.1. Materials

**Figure 1:**
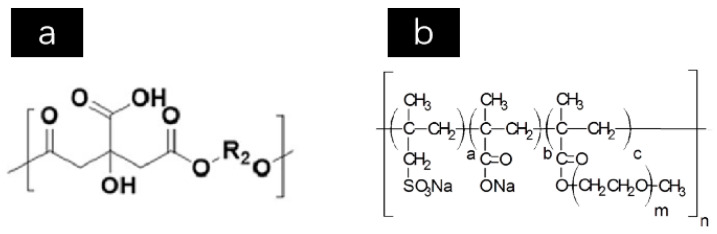
The chemical composition. (**a**) SAP. (**b**) Polycarboxylate superplasticizer.

**Figure 2:**
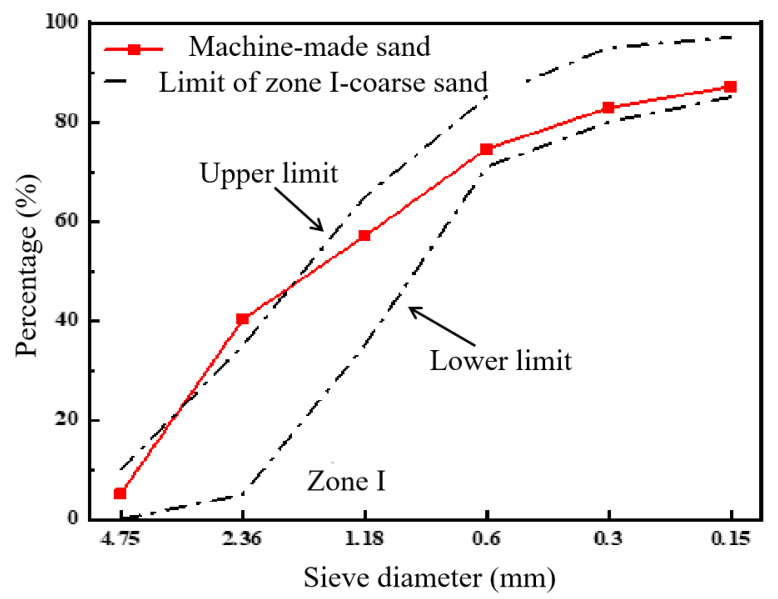
Machine-made sand grading curve.

**Figure 3:**
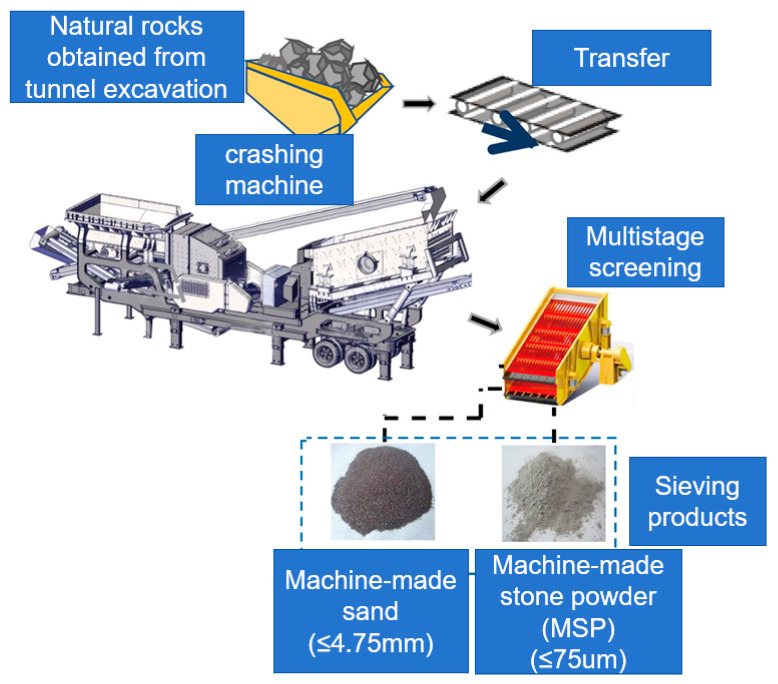
The preparation process of machine-made sand and MSP.

PO 42.5 ordinary silicate cement was produced by the Crane Forest brand, Zhenjiang City, Jiangsu Province, China. The cement exhibited a density of 3180 kg/m^3^ and a specific surface area of 368.6 m^2^/kg, and the mineral composition is shown in Table 1 as follows. The superabsorbent polymer (SAP), provided by a chemical plant in Jiangsu Province, China, had the characteristics detailed in Table 2. The chemical composition of SAP is shown in Figure 1a. The recycled coarse aggregate, with size ranging from 5 to 20 mm, was produced by waste concrete. The fine aggregate was divided into river sand and machine-made sand. The river sand, categorized as medium sand, was sourced from a building materials factory in Jiangsu Province, China. The machine-made sand was made from natural rocks generated during the excavation of tunnels by Puyan Expressway Co., Ltd., Sanming, China, and its grading curve is depicted in Figure 2. The grading interval of machine-made sand was in Zone I (coarse sand zone), the cumulative percentage was 87%, and the rest was the stone powder (MSP) less than 0.075 mm which was generated during the production of machine-made sand (Figure 3). The types and composition of minerals in the MSP are shown in Table 3. Polycarboxylate superplasticizer produced by Shanghai Chenqi Chemical Technology Co., Ltd. in Shanghai, China. The polycarboxylate superplasticizer had a water reduction rate less than 16%, and the chemical composition of the polycarboxylate superplasticizer is shown in Figure 1b. The dosage of polycarboxylate superplasticizer, which acted as the water-reducing agent, was set to 0.2% of the total weight of the cement.

### 2.2. Orthogonal Design for the Mix Proportions of Recycled Concrete

Recycled concrete with mixture of SAP and MSP was tested by the orthogonal test method of two factors and four levels. The SAP (0%, 0.08%, 0.16%, 0.24%) and MSP (0%, 3%, 6%, 9%) content was based on the cement mass percentage substituted. Referring to the common concrete mix proportion design code, a total of 16 sets of mix proportions were designed (Table 4). The sixteen samples were labeled as ‘SxMy’, where x and y represent the amount of SAP and MSP, respectively. There are three identical specimens with each sample.

### 2.3. Test Methodology

**Figure 4:**
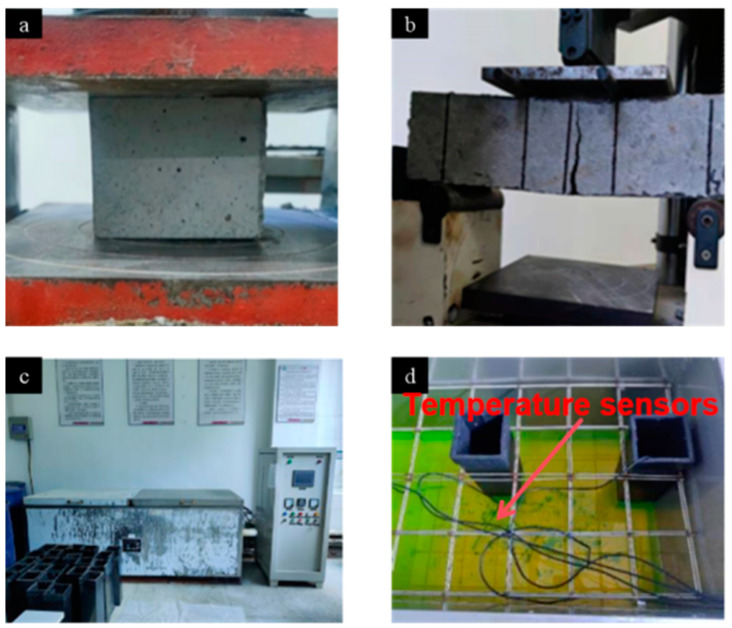
Mechanical test: (**a**) Compressive strength test. (**b**) Flexural compressive strength test. (**c**) CABR-HDK9 rapid freezing–thawing test machine of concrete. (**d**) Layout of temperature sensors.

This study focused on investigating the effect of the freeze–thaw cycle environment on the mechanical properties of recycled concrete. According to the standard for test methods of concrete physical and mechanical properties, the specimens with corresponding sizes were prepared, as shown in Table 5; three identical specimens were taken from each sample. The mechanical test was performed after 28 days of curing in a standard SHBY-40B curing box (Zhejiang Chenxin Test Machine Manufacturing Co., Ltd., Shaoxing, China). Mechanical property tests involved the measurement of the compressive strength and flexural strength of concrete cubes as plotted in Figure 4a,b. Placing the test block pending test into the SYE-2000 type pressure test (Hebei Sanyu Test Machine Manufacturing Co., Ltd., Cangzhou, China), we started the pressure tester and loaded it with 6 kN/s speed. After the specimen was damaged, we promptly recorded the compressive test data and turned off the pressure tester, then calculated the average of the three specimens. Placing the test specimen into WE-300B digital universal material testing machine (Wuxi Lushitong Test Machine Manufacturing Co., Ltd., Wuxi, China), the upper pressure plate did not touch the specimen; we opened the oil feed valve, the oil cylinder rose slowly, we pressed the “Zero” key to remove the tare, and then pressed a certain loading speed until the test specimen ruptures. We closed the oil feed valve, opened the oil return valve unloading, and recorded the flexural test data in time, taking the average of the three specimens. A total of 200 freeze–thaw cycles were applied in the CABR-HDK9 rapid freezing–thawing equipment (Beijing Jianyan Kunlun Technology Co., Ltd., Beijing, China) as shown in Figure 4c. The minimum temperature during the cycle was set to −18 ± 2 °C and the maximum temperature was set to 5 ± 2 °C. The temperature was controlled by the three temperature sensors in the freeze–thaw box, as shown in Figure 4d. The range of the temperature sensor indicates were controlled within 2 °C.

### 2.4. Orthogonal Experimental Range Analysis

The orthogonal experimental range analysis was designed for evaluation of the influence of SAP and MSP in mechanical properties, e.g., compressive strength, flexural strength. The value of range R (Equation (2)) was the difference between the maximum value and the minimum value of the test result $\bar{k_{ij}}$ (Equation (1)) at each factor *j*. Larger values of *R* represented the larger influence of the factor on the mechanical properties.

(1) $$\bar{k_{ij}}={\frac{1}{n}k}_{ij}$$

(2) $$R=\max\left\{\bar{k_{ij}}\right\}-\min\left\{\bar{k_{ij}}\right\}$$

where *K*_ij_ is the sum of the mechanical property test results of factor *j* with *i* level in the orthogonal table, and *n* is the number of parameter factors.

### 2.5. Strength Loss

In order to study the frost resistance of concrete under freeze–thaw cycling, the macroscopic mechanical properties such as compressive strength and flexural strength can be analyzed. The lower the strength loss before and after the freeze–thaw cycle, the better the freezing resistance of the concrete structure. The strength loss of each sample was calculated as Equation (3).

(3) $$f=\frac{f1-f2}{f1}$$

where *f*_1_ is the strength before freeze–thaw (MPa), and *f*_2_ is the strength after freeze–thaw (MPa).

## 3. Analysis and Discussion

### 3.1. Analysis of Compressive Strength Test Results

Table 6 described the compressive strength of 16 recycled concrete samples before and after freeze–thaw cycles and their strength loss. Table 7 presented the orthogonal experimental range analysis of compressive strength. Before freeze–thaw, the R value of SAP (6.28) is greater than that of MSP (4.45). The value R (6.58) of SAP after freeze–thaw cycles is lower than that of MSP (8.10). It could be noted that the influence of SAP on compressive strength is larger than that of MSP before freeze–thaw cycles, But after freeze–thaw cycles, the situation is the opposite, and the MSP content has a greater impact.

From the Table 7, among the four levels of SAP (0%, 0.08%, 0.16%, 0.24%), the optimal level of compressive strength before and after freeze–thaw cycles is 0.08% due to the larger $\bar{k_{ij}}$ value. Among the four levels of MSP (0%, 3%, 6%, 9%), the optimal level before freeze–thawing is 9%, and after freeze–thaw cycles is 6%. This shows that the more MSP affects the compressive strength of the structure in a freeze–thaw environment, destroys its internal structure, leads to a greater reduction in mechanical properties, and a large difference in R value before and after freeze–thaw.

**Figure 5:**
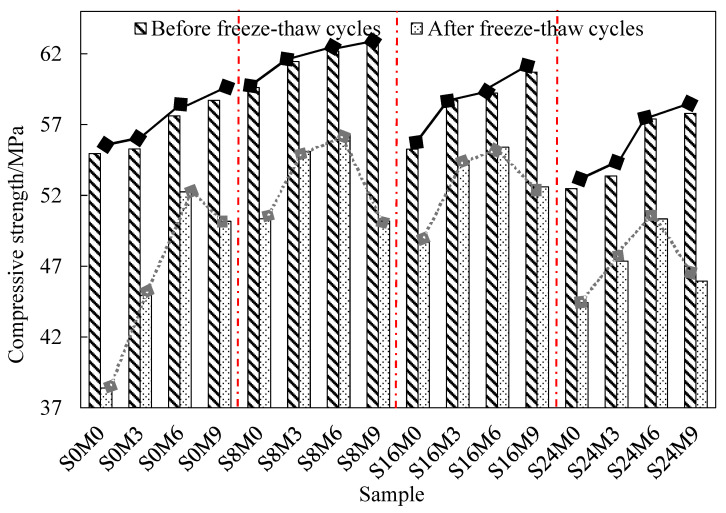
Compressive strength before and after freeze–thaw cycles (same SAP).

Table 6 and Figure 5 show the compressive strength of recycled concrete before and after freeze–thaw cycles. Generally, freeze–thaw cycles may cause concrete to lose moisture and increase structural voids [57]. Therefore, there is a loss of compressive strength after freeze–thaw cycles, and the minimum strength loss all is content of SAP 0.16% or MSP 6%. From Table 6, when the MSP content is the same, with the increase in SAP content, compressive strength increases first and then decreases before and after freeze–thaw cycles, which is consistent with the Babatunde et al. [53]. As can be seen from Figure 5, when SAP content is the same, with the increase in MSP content, compressive strength always increases before freeze–thaw, but after freeze–thaw it increases first and then decreases, which is related to the addition of excessive MSP. Therefore, there is an optimal content of SAP and MSP.

The maximum compressive strength before freeze–thaw is 62.88 MPa (Sample: S8M9), the maximum compressive strength after freeze–thaw is 56.34 MPa (Sample: S8M6), and the minimum compressive strength loss before and after freeze–thaw is 6% (Sample: S16M6). The compressive strength loss of S8M9 before and after freeze–thaw is 20%, which may have contributed to the excessive MSP hindering the hydration reaction and causing an increase in internal defects in the structure. Compared with S8M6 and S16M6, the compressive strength of S16M6 decreases by 4.76% before freeze–thaw and 1.69% after freeze–thaw, and the compressive strength of S16M6 after freeze–thaw is second only to S8M6. This is mainly because the increase in SAP content reduces the porosity of recycled concrete, increases the compactness, and improves the frost resistance [58]. As SAP content continues to increase, the compressive strength of S24M6 after freezing and thawing decreases by 9.15% compared with S16M6. This is because excessive SAP will remain in the concrete and develop into a larger void, which affects the freeze resistance of concrete [58]. Therefore, it could be considered that recycled concrete with best frost resistance is the sample S16M6 (SAP 0.16%, MSP 6%), and its compressive strength is 44% higher than S0M0.

### 3.2. Analysis of Flexural Strength Test Results

Table 8 describes the flexural strength of 16 recycled concrete samples before and after freeze–thaw cycles and their strength loss. Table 9 shows the orthogonal experimental range analysis of flexural strength. The R-value analysis of flexural strength is consistent with that of compressive strength. The R-value is SAP > MSP before freeze–thaw and SAP < MSP after freeze–thaw. This may be due to the strong ability of SAP to absorb water and retain water. Before the freeze–thaw cycle, SAP can absorb water to form a solid gel and increase the mechanical properties of the structure. However, after the freeze–thaw cycle, the strength of the structure will be affected because of the formation of ice by water [59]. The micro-aggregate effect of MSP enhances the density of concrete, reduces the porosity of concrete, and better hinders the entry of external water. Therefore, the more MSP is mixed in the freeze–thaw environment, the greater the improvement of structural strength [25]. Therefore, of particular note is that the amount of MSP of recycled concrete in the freeze–thaw cycle environment.

From the Table 9, among the four levels of SAP (0%, 0.08%, 0.16%, 0.24%), the optimal level of flexural strength before and after freeze–thaw cycles is 0.16% due to the larger $\bar{k_{ij}}$ value. Among the four levels of MSP (0%, 3%, 6%, 9%), the optimal level before and after freeze–thaw cycles is 6%.

**Figure 6:**
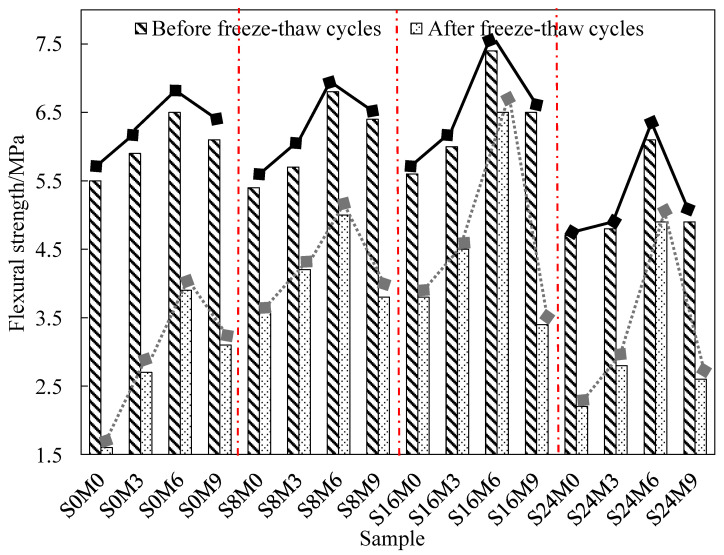
Flexural strength values of concrete before and after freeze–thaw cycles (Same SAP).

As shown in Table 8 and Figure 6, the flexural strength of recycled concrete is evaluated both before and after freeze–thaw cycles. When the MSP content is kept constant (Table 8), an increase in SAP leads to an initial increase in flexural strength, followed by a decrease, with the highest strength observed at the third level of SAP (0.16%). Similarly, when the SAP content is held constant (Figure 6), increasing the MSP content also results in an initial increase in flexural strength, which then decreases, with the maximum strength occurring at the third level of MSP (6%).

The maximum flexural strength before freeze–thaw is 7.4 MPa (Sample: S16M6), the maximum compressive strength after freeze–thaw is 6.5 MPa (Sample: S16M6), and the minimum compressive strength loss before and after freeze–thaw is 12% (Sample: S16M6). The analysis of flexural strength further proves that S16M6 (SAP 0.16%, MSP 6%) is the best admixture ratio of recycled concrete in freeze–thaw environment.

### 3.3. Regression Analysis

In order to further analyze the relationship between compressive strength and flexural strength before and after 200 freeze–thaw cycles, three sets of strength values under the optimal mix proportion (S16M6: 0.16% SAP and 6% MSP) are selected to establish the linear regression model:

$$y=0.9433x-0.4736{\;R}^{2}=0.99$$

where *x* is the strength value before freeze–thaw cycles, and *y* is the strength model predicted value after freeze–thaw cycles.

The strength value of S16M6 before and after 200 freeze–thaw cycles and the model prediction of value are shown in Table 10. The standard deviation of regression analysis is 0.1. This regression model is helpful to predict the compressive and flexural strength of recycled concrete mixed with SAP after freezing and thawing.

## 4. Conclusions

This study investigated the mechanical properties and frost resistance of recycled concrete with the combined incorporation of SAP (0%, 0.08%, 0.16%, and 0.24%), and MSP (0%, 3%, 6%, and 9%). The optimal mix proportions for performance under freeze–thaw conditions were determined, and a linear regression model was established to predict the mechanical strength before and after freeze–thaw cycles. The detailed conclusions of this study were as follows:

1. Based on the extreme value analysis of the orthogonal test, it was found that, for both compressive strength and flexural strength, the R-value of SAP was greater than that of MSP before freeze–thaw cycles, while after freeze–thaw cycles, the R-value of MSP exceeded that of SAP. We proposed that the influence of SAP on the mechanical properties was dominant in the normal environment (before freeze–thaw) due to its higher water absorbency and water retention performance. However, after freeze–thaw, the outer water was blocked by MSP through the micro-aggregate effect resulting in the improved mechanical properties of recycled concrete. This indicated that the amount of MSP should be given special attention when designing recycled concrete for freeze–thaw environments. According to the larger $\bar{k_{ij}}$ value, the optimal levels of the $\bar{k_{ij}}$ value for compressive strength were SAP at 0.08% and MSP at 6% (before freeze–thaw) or 9% (after freeze–thaw), while the optimal levels of the $\bar{k_{ij}}$ value for flexural strength were SAP at 0.16% and MSP at 6%.
2. From the perspective of compressive strength, as the SAP content increased, compressive strength first increased and then decreased, both before and after freeze–thaw cycles. With increasing MSP content, compressive strength consistently increased before freeze–thaw cycles, and first increased and then decreased after freeze–thaw cycles. The maximum compressive strength before freeze–thaw was observed in S8M9, and after freeze–thaw in S8M6. The minimum strength loss before and after freeze–thaw was found in S16M6. After freeze–thaw cycles, the excessive MSP in S8M9 resulted in increasing defects within the structure, caused a large loss of structural strength, and reduced frost resistance. As the SAP content increased, the density of the structure increased, and the frost resistance improved. However, excessive SAP formed voids in the concrete and reduced the frost resistance of the structure. The mix with the best frost resistance was S16M6 (SAP 0.16%, MSP 6%).
3. From the perspective of flexural strength, flexural strength also followed a trend of first increasing and then decreasing with the increase in both SAP and MSP content, both before and after freeze–thaw cycles. The S16M6 mix consistently showed the highest flexural strength and the smallest strength loss, both before and after freeze–thaw cycles.
4. A linear regression model was established for the compressive and flexural strengths of recycled concrete with the optimal mix ratio of SAP 0.16% and MSP 6%, before and after freeze–thaw cycles. This model provides a valuable reference for predicting the behavior of recycled concrete mixed with SAP in freeze–thaw environments.

## Figures and Tables

**Table 1:** Mineral composition of the cement (wt%).

Mineral Composition	C_3_S	C_2_S	C_3_A	C_4_AF	Gypsum	Quartz	Periclase	K_2_SO_4_	Free Lime
Content	60.2	14.8	3.8	12.7	3.5	0.1	0.1	0.9	1.2

**Table 2:** Characteristics of SAP.

	Water Absorption Ratio (g/g)	Brine Absorption Ratio (g/g)	Pressure Water Absorption Ratio (g/g)	Packing Density (g/mL)	pH
Numeric value	400–500	60	26	0.65–0.85	6.2

**Table 3:** Mineral composition of the MSP (wt%).

	Quartz	Potassium Feldspar	Plagioclase	Calcite	Dolomite
MSP	3.2	0.3	0.4	80.1	15.1

**Table 4:** Mixing ratio of concrete (kg/m^3^).

Sample	SAP (%)	MSP (%)	Cement	River Sand	Machine-Made Sand	Coarse Aggregate	Water	Polycarboxylate Superplasticizer
S0M0	0.00	0	455.0	141	564.2	1057.8	182.00	0.91
S8M0	0.08	0	454.7	141	564.2	1057.8	191.09	0.91
S16M0	0.16	0	454.3	141	564.2	1057.8	191.09	0.91
S24M0	0.16	0	453.9	141	564.2	1057.8	191.08	0.91
S0M3	0.00	3	441.4	141	564.2	1057.8	182.00	0.91
S8M3	0.08	3	441.0	141	564.2	1057.8	190.82	0.91
S16M3	0.16	3	440.6	141	564.2	1057.8	190.81	0.91
S24M3	0.24	3	440.1	141	564.2	1057.8	190.80	0.91
S0M6	0.00	6	427.7	141	564.2	1057.8	182.00	0.91
S8M6	0.08	6	427.3	141	564.2	1057.8	190.55	0.91
S16M6	0.16	6	427.0	141	564.2	1057.8	190.54	0.91
S24M6	0.24	6	426.6	141	564.2	1057.8	190.53	0.91
S0M9	0.00	9	414.1	141	564.2	1057.8	182.00	0.91
S8M9	0.08	9	413.7	141	564.2	1057.8	190.27	0.91
S16M9	0.16	9	413.3	141	564.2	1057.8	190.27	0.91
S24M9	0.24	9	413.0	141	564.2	1057.8	190.26	0.91

**Table 5:** Specimen specifications.

No.	Test Content	Specimen Size/mm
1	Compressive strength	100 × 100 × 100
2	Flexural strength	100 × 100 × 400

**Table 6:** Compressive strength and strength loss before and after freeze–thaw cycles (Same MSP).

Sample	Factor		Compressive Strength (MPa)		Strength Loss (%)
	SAP (%)	MSP (%)	*f*_1_	*f*_2_	
S0M0	0.00	0	54.95	38.38	30
S8M0	0.08	0	59.61	50.35	16
S16M0	0.16	0	55.26	48.80	12
S24M0	0.24	0	52.47	44.41	15
S0M3	0.00	3	55.27	44.94	19
S8M3	0.08	3	61.45	55.10	10
S16M3	0.16	3	58.73	54.44	7
S24M3	0.24	3	53.37	47.35	11
S0M6	0.00	6	57.62	52.25	9
S8M6	0.08	6	62.19	56.34	9
S16M6	0.16	6	59.23	55.40	6
S24M6	0.24	6	57.39	50.33	12
S0M9	0.00	9	58.71	50.16	15
S8M9	0.08	9	62.88	50.18	20
S16M9	0.16	9	60.71	52.60	13
S24M9	0.24	9	57.78	45.94	20

**Table 7:** Analysis on the range of compressive strength.

	SAP (%)	MSP (%)
$\bar{k_{1j}}$-1	56.64	55.57
$\bar{k_{2j}}$-1	61.53	57.21
$\bar{k_{3j}}$-1	58.48	59.11
$\bar{k_{4j}}$-1	55.25	60.02
$\bar{k_{1j}}$-2	46.43	45.49
$\bar{k_{2j}}$-2	52.99	50.46
$\bar{k_{3j}}$-2	52.81	53.58
$\bar{k_{4j}}$-2	47.01	49.72
R-1	6.28	4.45
R-2	6.56	8.10

(Ps: $\bar{k_{ij}}$-1, R-1 represented the degree of influence of SAP and MSP on recycled concrete before freeze–thaw cycles; $\bar{k_{ij}}$-2, R-2 indicated the degree of influence after 200 freeze–thaw cycles).

**Table 8:** Flexural strength and strength loss before and after freeze–thaw cycles (Same MSP).

Sample	Factor		Flexural Strength (MPa)		Strength Loss (%)
	SAP (%)	MSP (%)	*f*_1_	*f*_2_	
S0M0	0.00	0	5.5	1.6	71
S8M0	0.08	0	5.4	3.6	33
S16M0	0.16	0	5.6	3.8	32
S24M0	0.24	0	4.7	2.2	53
S0M3	0.00	3	5.9	2.7	54
S8M3	0.08	3	5.7	4.2	26
S16M3	0.16	3	6.0	4.5	25
S24M3	0.24	3	4.8	2.8	42
S0M6	0.00	6	6.5	3.9	40
S8M6	0.08	6	6.8	5.0	26
S16M6	0.16	6	7.4	6.5	12
S24M6	0.24	6	6.1	4.9	20
S0M9	0.00	9	6.1	3.1	41
S8M9	0.08	9	6.4	3.8	49
S16M9	0.16	9	6.5	3.4	48
S24M9	0.24	9	4.9	2.6	47

**Table 9:** Analysis on the range of flexural strength.

	SAP (%)	MSP (%)
$\bar{k_{1j}}$-1	6.08	5.30
$\bar{k_{2j}}$-1	6.00	5.60
$\bar{k_{3j}}$-1	6.38	6.70
$\bar{k_{4j}}$-1	5.13	5.98
$\bar{k_{1j}}$-2	3.00	2.80
$\bar{k_{2j}}$-2	3.98	3.55
$\bar{k_{3j}}$-2	4.55	5.08
$\bar{k_{4j}}$-2	3.13	3.23
R-1	1.25	1.40
R-2	1.55	2.28

(Note: $\bar{k_{ij}}$-1, R-1, $\bar{k_{ij}}$-2, R-2 are consistent with Table 4).

**Table 10:** Strength value of S16M6.

Strength Value	Before 200 Freeze–Thaw Cycles (MPa)	Experimental Values after 200 Freeze–Thaw Cycles (MPa)	Model Predicted Value after 200 Freeze–Thaw Cycles (MPa)
Compressive strength	59.230	55.400	55.398
Flexural strength	7.400	6.500	6.507

## Data Availability

All data generated or analyzed in this research were included in this published article. Additionally, readers can access all data used to support the conclusions of the current study from the corresponding author upon request.
